# F-Box Protein 43, Stabilized by N6-Methyladenosine Methylation, Enhances Hepatocellular Carcinoma Cell Growth and Invasion via Promoting p53 Degradation in a Ubiquitin Conjugating Enzyme E2 C-Dependent Manner

**DOI:** 10.3390/cancers15030957

**Published:** 2023-02-02

**Authors:** Huijun Zhou, Chong Zeng, Jie Liu, Haijun Luo, Wei Huang

**Affiliations:** 1 Department of Gastroenterology and Urology, Hunan Cancer Hospital/The Affiliated Cancer Hospital of Xiangya School of Medicine, Central South University, Changsha 410083, China; 2 Department of Medicine, The Seventh Affiliated Hospital, Hengyang Medical School, University of South China, Changsha 410004, China; 3 Department of Pathology, The Affiliated Changsha Central Hospital, Hengyang Medical School, University of South China, Changsha 410004, China; 4 Department of Oncology, Xiangya Hospital, Central South University, Changsha 410083, China; 5 Research Center of Carcinogenesis and Targeted Therapy, Xiangya Hospital, Central South University, Changsha 410083, China; 6 National Clinical Research Center of Geriatric Disorders, Xiangya Hospital, Central South University, Changsha 410083, China

**Keywords:** HCC, FBXO43, m6A modification, p53, UBE2C

## Abstract

**Simple Summary:**

The role of FBXO43 and the underlying mechanism in cancer are largely unknown. In the present study, we investigated the expression, clinical and prognostic value, and functions of FBXO43 in HCC. The results demonstrated that FBXO43 was upregulated in HCC, was positively associated with advanced pathological stages and poor prognosis, and increased cell proliferation and invasion. Moreover, findings revealed that METTL3 and IGF2BP2 mediated m6A modification stabilized FBXO43, which facilitates the malignant progression of HCC. Mechanistically, FBXO43 exerts its oncogenic role in HCC by promoting ubiquitin-dependent proteasomal degradation of p53 through UBE2C upregulation.

**Abstract:**

The roles of F-box protein 43 (FBXO43) in carcinogenesis have been rarely revealed. The present study investigates the expression, function, and underlying mechanism of FBXO43 in hepatocellular carcinoma (HCC). Firstly, the expression and clinical significance of FBXO43 in HCC were investigated bioinformatically and experimentally using online omics data and local tissue samples. The role of N6-methyladenosine modification (m6A) of mRNA in regulating FBXO43 expression and the effects of m6A/FBXO43 axis alteration on cell proliferation and invasion were investigated further. Moreover, the underlying mechanism of the oncogenic FBXO43 was also explored. The results demonstrated that FBXO43 was significantly upregulated in HCC and was positively correlated with advanced progression and poor prognosis in patients. METTL3 and IGF2BP2 expressions were positively correlated with FBXO43 expression and served as the writer and reader of FBXO43 m6A, respectively, which stabilized and upregulated FBXO43 mRNA in HCC. FBXO43 silencing significantly reduced cell proliferation and invasion, and ectopic expression of FBXO43 could significantly restore the inhibitory effects caused by METTL3 and IGF2BP2 depletion in HCC cells. Mechanistically, FBXO43 depletion reduced the expression of UBE2C, a p53 ubiquitin-conjugating enzyme, suppressed proteasomal degradation of p53, and thus inhibited cell proliferation and invasion in HCC. In summary, the present study revealed that METTL3/IGF2BP2 mediated m6A contributed to the upregulation of FBXO43 that promoted the malignant progression of HCC by stimulating p53 degradation in a UBE2C-dependent manner, highlighting the promising application of FBXO43 as a target in HCC treatment.

## 1. Introduction

Hepatocellular carcinoma (HCC) is the most common type of primary liver tumor. HCC is the fifth most common type of cancer and the third leading cause of cancer-related death [1]. Because early HCC symptoms are indistinct, most HCC patients have been diagnosed at late stages and have lost the chance of curative treatment [1]. Therefore, the unsatisfactory clinical outcomes of HCC demand more in-depth research on the pathogenesis of HCC. Identifying HCC drive genes and elucidating the underlying mechanisms are critical steps toward identifying more effective therapeutic targets and ultimately improving treatment outcomes.

F-box protein 43 (FBXO43), also known as endogenous meiotic inhibitor 2 (EMI2), is a member of the F-box protein family, characterized by an approximately 40-amino acid F-box motif [2]. As a critical component of cytostatic factor, FBXO43 is well-known for establishing and maintaining oocytes arrest at the second meiotic metaphase until fertilization by inhibiting the anaphase-promoting complex/cyclosome (APC/C) ubiquitin ligase [2,3]. FBXO43 has also been implicated in regulating multiciliated cell differentiation [4]. Bioinformatical analyses have recently validated the aberrant expression, prognostic values, and functions of FBXO43 in breast cancer, as well as the potential oncogenic roles of FBXO43 in gastric cancer and HCC [5,6,7,8]. These findings imply the critical roles of FBXO43 in tumorigenesis and highlight the importance and necessity of further studies focusing on FBXO43 in cancer.

The post-transcriptional modification N6-methyladenosine (m6A) is important for RNA stability, translation, alternative splicing, and subcellular localization [9]. Writers (METTL3, METTL14, METTL16, and WTAP), readers (IGF2BPs and YTHDFs), and erasers (FTO, ALKBH5) catalyze m6A [9]. A dysregulation of m6A modification affects the transcriptome and proteome, regulating a wide range of biological functions [10]. Numerous studies have demonstrated that m6A is important in carcinogenesis, causing cancer aberrant gene expression [9,10]. It is unclear whether m6A regulates the expression of FBXO43.

The present study investigated the expression and function of FBXO43 in HCC and the mechanisms underlying its upregulation. The findings demonstrated that FBXO43 was upregulated in HCC, predicted poor prognosis, and promoted cell proliferation and invasion. It is worth noting that IGF2BP2 and METTL3 could both write and recognize FBXO43 mRNA, resulting in increased malignant progression of HCC. FBXO43 depletion decreased UBE2C expression and upregulated p53 by inhibiting its ubiquitination-mediated degradation in HCC. The present study primarily revealed the upregulation and oncogenic roles of FBXO43 in HCC and the mechanisms underlying these roles, highlighting the promising potential of FBXO43 as a treatment for HCC.

## 2. Materials and Methods

### 2.1. Cell Lines, Small Interfering RNAs (siRNAs), and Plasmids

HCC cell lines, including HCCLM3, HepG2, HCC8910, and Huh7, and normal hepatic cell line LO2, were obtained from Procell (Wuhan, China) and cultured in Dulbecco’s Modified Eagle Medium (DMEM, # C3110-0500, VivaCell, Shanghai, China) supplemented with 10% fetal bovine serum (FBS, # 164210-50, Procell, Wuhan, China) in a humidified cell incubator at 37 °C with 5% CO_2_. Small interfering RNAs (siRNAs) targeting METTL3, IGF2BP2, FBXO43, and siNC (negative control) were purchased from Sangon Biotech (Shanghai) Inc. (Shanghai, China). Supplementary Table S1 contains a list of siRNAs sequences. pHG-CMV-FBXO43 and pHG-CMV-p53 plasmid were purchased from Honor Gene Inc. (Changsha, China). The pENTER-UBE2C and control plasmid were obtained from WZ Bioscience Inc. (Shandong, China). According to the manufacturer’s instructions, the plasmids and siRNAs were introduced into HCC cells using Lipo8000^TM^ reagent (#C0533, Beyotime, Shanghai, China). 

### 2.2. Tissue Samples

A total of 40 fresh liver tissues, including twenty tumor tissues and twenty normal liver tissues (>2 cm from the resection margin), were obtained from the surgical tissues of patients with primary HCC, before receiving any other therapies such as radio-/chemo-therapy, targeted therapy, and immunology therapy, at Hunan Cancer Hospital. The fresh tissues were divided into two parts. One part was subjected to RNA extraction and the other part was fixed with formalin and embedded in paraffin. An HCC tissue chip containing 80 tumor tissues and paired adjacent normal tissues (#LVC1607) was purchased from Shanghai Superbiotek Pharmaceutical Technology Inc. (Shanghai, China). Metastatic hepatocellular carcinoma and other pathological types of liver cancer such as intrahepatic cholangiocarcinoma and fibrolamellar HCC were not included in the study. Patients signed informed consent forms before surgery, and the study was approved by the Ethical Committee of Hunan Cancer Hospital.

### 2.3. Online Analysis

Level 3 of HCC RNA-sequence data and the paired clinical data from TCGA (The Cancer Genome Atlas, https://portal.gdc.cancer.gov/, accessed on 10 October 2022) were used to investigate the expression, clinical relationship, and prognostic value of FBXO43, as well as the correlations between FBXO43 and m6A regulators by R (version 3.6.3) as described previously [11]. To perform expression, correlation, and prognostic analyses, ENCORI (The Encyclopedia of RNA Interactomes), Kaplan–Meier Plotter (Liver cancer RNA-seq), UALCAN, and GENT2 online services were used [12,13,14,15].

### 2.4. IHC Assay

Using the HCC tissue chip mentioned above, the levels of FBXO43 in HCC and adjacent normal tissues were determined using the IHC assay described previously [15]. Rabbit anti-FBXO43 polyclonal antibody (#55176-1-AP, Proteintech, IL, USA) was used at 1:100 dilution. The co-expression of FBXO43 (#55176-1-AP, Proteintech, IL, USA), METTL3 (#15073-1-AP, dilution:1:200, Proteintech, IL, USA), IGF2BP2 (#11601-1-AP, dilution:1:200, Proteintech, IL, USA), UBE2C (#D124141, dilution:1:50, BBI, Shanghai, China), and p53 (#sc-126, dilution:1:50, Santa Cruz, TX, USA) were also detected in twenty paraffined-HCC tissues by IHC using slides of consecutive serial sections. Semi-quantitative analysis of the IHC assay was performed based on staining intensity and area, as mentioned previously [16]. Briefly, a total of three staining intensities were classified: absent staining as 0, weak staining as 1, moderate staining as 2, and strong staining as 3. Based on the number of stained cells (examined in at least 500 cells), we classified them as non-stained = 0, 30% of stained cells = 1, 30–60% = 2, and >60% = 3. Each tissue’s staining score was calculated by adding the area and intensity scores. A combined staining score of ≤3 indicated low expression, and >3 indicated high expression.

### 2.5. Quantitative Reverse Transcription Polymerase Chain Reaction (qRT-PCR)

qRT-PCR was performed as previously described [16]. Briefly, total RNAs from tissues and cells were isolated using TrizolTM reagent (#5301100, SIMGEN, Hangzhou, China) and reverse transcribed into cDNA by Hifair^®^ III 1st Strand Cdna Synthesis SuperMix for Qpcr (Gdna digester plus) (#11141ES10, YEASEN, Shanghai, China) according to the manufacturer’s instructions. A Hieff^®^ Qpcr SYBR Green Master Mix (#11201ES03, YEASEN, Shanghai, China) was used to measure the relative levels of FBXO43, FBXO43 heterogeneous nuclear RNA (FBXO43 hnRNA), METTL3, and IGF2BP2 with GAPDH as a control, using delta-delta Ct method (2^−ΔΔCt^). Supplementary Table S1 contains the primer sequences. 

### 2.6. Methylated RNA Immunoprecipitation Quantitative Polymerase Chain Reaction (MeRIP-qPCT)

Methylated RNA Immunoprecipitation (MeRIP) was used to validate the m6A modification of FBXO43 using the BersinBio^TM^ MeRIP Kit (#Bes5203, BersinBio, Guangzhou, China) following the manufacturer’s instruction. Briefly, a 100 μg fragmented RNA sample was treated with 4 g anti-m6A antibody and immunoglobulin G (IgG) in IP buffer for 4 h at 4 °C with protease and Rnase inhibitor. Then, 30 μL of washed protein A/G magnetic beads were added to IP buffer containing M6A/IgG and RNA mixture and incubated at 4 °C for 1 h. Phenol/Chloroform/Isoamyl alcohol (25:24:1, # P1011, Solabio, Beijing, China) extractions were used to elute and purify the precipitated RNAs. The eluted RNAs were transcribed into cDNA to perform qRT-PCR assays. The relative level of m6A-FBXO43 was analyzed using the follow methods: %input = 2^(−ΔCt normalized RIP)^, ΔCt nommalized RIP = (Average CtRIP − Average CtInput − log_2_(input dilution factor)). Supplementary Table S1 displays the primer sequences.

### 2.7. RNA Immunoprecipitation (RIP)

The Magna RIP RNA-Binding Protein Immunoprecipitation Kit (#17-700, Millipore, MA, USA) was used to perform RIP according to the manufacturer’s instructions. The magnetic beads were conjugated with IGF2BP2 (#11601-1-AP, Proteintech, IL, USA) or IgG antibodies for 30 min. The cell lysate was then rotated with magnetic beads overnight at 4 °C. After proteinase K digestion and washing; bound RNAs were extracted with Phenol/Chloroform/Isoamyl alcohol (125:24:1, #P1025, Solabio, Beijing, China). The relative interaction between IGF2BP2 protein and FBXO43 mRNA was determined using qRT-PCR. The relative level of m6A-FBXO43 was analyzed using the follow methods: %input = 2^(−ΔCt normalized RIP)^, ΔCt nommalized RIP = [Average CtRIP − Average CtInput − log_2_(input dilution factor)].

### 2.8. T7 Biotin Labeled RNA Synthesis and RNA Pulldown

Ribo^TM^ RNAmax-T7 Biotin Labeled RNA Synthesis Kit (#C11002-2, RiboBio, Guangzhou, China) was used to prepare biotin-labeled RNA FBXO43 mRNA probes according to the manufacturer’s instructions. Then, an RNA pulldown assay was performed using the BersinBio^TM^ RNA pulldown Kit (#Bes5102, BersinBio, Guangzhou, China) through standard procedure. Briefly, RNA probes labeled with biotin were conjugated to streptavidin-coated magnetic beads at room temperature for 30 min. RNA-conjugated magnetic beads were then incubated for 2 h at room temperature with cell lysates devoid of nucleic acids to capture binding proteins. Finally, the bound proteins were eluted and analyzed using western blotting. Supplementary Table S1 displays the primer sequences.

### 2.9. RNA Stability Assays

RNA stability assays were performed as previously described [17]. HCC cells were treated with actinomycin D (#HY-17559,10 g/mL, MCE, NJ, USA) for 0, 2, and 4 h. Following RNA isolation, qRT-PCR was performed to determine the relative expression of FBXO43, normalized to GAPDH expression. mRNA half-lives were estimated using linear regression analysis.

### 2.10. Western Blot (WB)

The Western blot (WB) was performed as previously described [16]. Briefly, RIPA (#WB3100, NCM Biotech, Suzhou, China) treated cell lysis was centrifuged at 4 °C to obtain total proteins. Proteins were denatured and electrophoresed on polyacrylamide gels with 12% sodium dodecyl sulfate before being transferred to polyvinylidene difluoride membranes. After blocking with 5% non-fat milk, the membrane was incubated with the primary antibodies, including rabbit anti-FBXO43, rabbit anti-IGF2BP2, anti-METTL3 (dilution: 1:500, 1:1000, 1:500; #55176-1-AP, #11601-1-AP, #15073-1-AP, Proteintech, IL, USA), rabbit anti-UBE2C and anti-p21 (dilution: 1:400, #D124141, #D120403, BBI, Shanghai, China), mouse-p53 (dilution: 1:100, #sc-126, Santa Cruz, TX, USA), rabbit-Cleaved-PARP-1 (dilution: 1:500, Immunoway, TX, USA) and rabbit anti-GAPDH antibody (dilution: 1:2000, #BS65656, Bioworld, Nanjing, China), respectively. After incubation with HRP-conjugated anti-rabbit and anti-mouse secondary antibodies (dilution: 1:3000, #D110058/D110098 BBI, Shanghai, China), the protein amounts were visualized using chemiluminescent HRP substrate (#SQ202, EpiZyme, Shanghai, China). The optical densities of Western blot bands were obtained using ImageJ analysis software.

### 2.11. Immunoprecipitation (IP)

The IP assay was performed as previously described [16]. Protein A/G Magnetic Beads (#HY-K0202, MCE, Junction, NJ, USA) were used for the IP assay. Briefly, the magnetic beads were washed and pre-conjugated with mouse anti-p53 (#sc-126, Santa Cruz, TX, USA) at 4 °C for 2 h. The magnetic beads conjugated with p53 antibodies were mixed with MG-132 (20Μm, # HY-13259, MCE, NJ, USA) treated cell lysis at 4 °C for 4 h. Finally, the precipitated proteins were eluted from the magnetic beads by boiling in loading buffer (#WB2001, NCM, Suzhou, China) for 7 min and subjected to WB analysis to detect the p53 ubiquitination using rabbit-anti Ubiquitin (E6K4Y) (#20326, CST, Chicago, IL, USA).

### 2.12. Luciferase Reporter Assay

A dual-luciferase reporter system assay was performed by the previously described procedure [17]. Briefly, the sequences containing predicted m6A sites, including 4 pieces of sequences with very high M6A confidence (Supplementary Table S2), for FBXO43 were synthesized and cloned into pmirGLO reporter plasmids) by Sangon Biotech (Shanghai, China) Co., Ltd. (Shanghai, China). The HCC cells were then co-transfected with pmirGLO-FBXO43 plasmids and siRNAs. After 48 h, luciferase activity in the cell lysate was measured using Steady-Glo^®^ Luciferase Assays (Promega, Fitchburg, WI, USA). Firefly luciferase activity was normalized by Renilla luciferase activity to reflect relative expression activity. otometer (BioTek, WI, USA), and growth curves were plotted based on each absorbance. The experiments were biologically repeated for threes times.

### 2.13. CCK-8 Assay

CCK-8 assay was performed by a previously described method to detect cell growth [16]. The cells were seeded at a density of 1 × 104 cells per well in a 96-well plate. The CCK-8 reagent (#HY-K0301, MCE, NJ, USA) was added to the medium for five days. After 1 h of incubation, the absorbance was measured at 450 nm using a spectrophotometer (BioTek, WI, USA), and growth curves were plotted based on each absorbance. The experiments were biologically repeated for threes times.

### 2.14. Plate Clone Formation Assay

Plate clone formation assay was performed as previously described [18]. Briefly, 1 × 103 cells per well were seeded into a 6-well plate and cultured for seven days. The cells were then fixed in methanol and stained with 0.5% crystal violet. The cell clones were photographed and counted for statistical analysis. The experiments were biologically repeated for threes times.

### 2.15. Transwell Invasion Assay

Transwell assay was carried out as previously described [18]. Briefly, 2.5 × 104 cells were seeded into a transwell chamber (#3422, Costar, ME, USA), and 750 mL of DMEM medium containing 5% FBS was poured into the lower well. After 20 h, the cells were fixed in methanol and stained with 0.5% crystal violet. After swabbing cells on the upper side of the membrane, the invasive cells on the upper side of the membrane were photographed and counted using an inverse microscope (Leica, Solms, Germany). The experiments were biologically repeated for threes times.

### 2.16. Statistical Analysis

The GraphPad Prism 8.0 software (MA, USA) was used for statistical analysis and chart plotting. The Student *t*-test or Fisher exact test was used for comparing the two groups and one-way ANOVA was used for comparing multiple groups. Correlation was determined using Pearson and Spearman correlation. The Kaplan–Meier method was used to draw survival curves, and the Log-rank test was used for the statistical analysis. *p* < 0.05 was considered statistically significant.

## 3. Results

### 3.1. A High Level of FBXO43 Is Associated with Malignant Progression and Poor Prognosis in HCC

**Figure 1:**
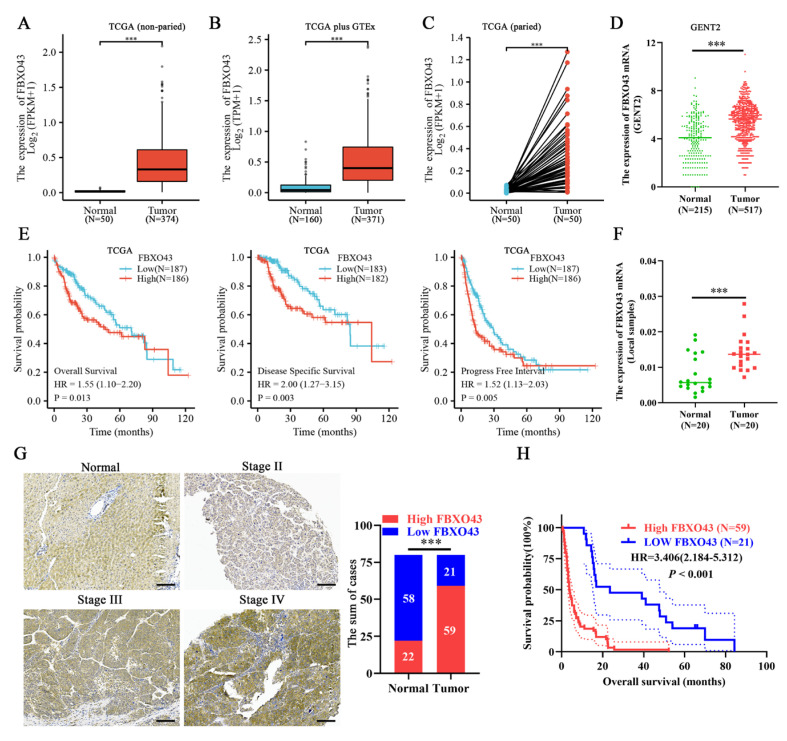
FBXO43 is upregulated and predicts poor prognosis in HCC. The online data indicating the expression of FBXO43 between normal liver and HCC tissues: (**A**) TCGA data (non-paired), (**B**) TCGA plus GTEx data (non-paired), (**C**) TCGA data (paired), and (**D**) GENT2 data (non-paired). (**E**) The TCGA data indicating the prognostic value of FBXO43 in HCC including OS, DSS, and PFI. (**F**) qRT-PCR indicating the expression of FBXO43 in local HCC tissues. (**G**) The representative pictures of FBXO43 staining in HCC and normal tissues. (**H**) The survival curve indicating the overall survival rate in HCC patients with low and high FBXO43 level. Scale bar 100 μm. ***, *p* < 0.001.

**Figure 2:**
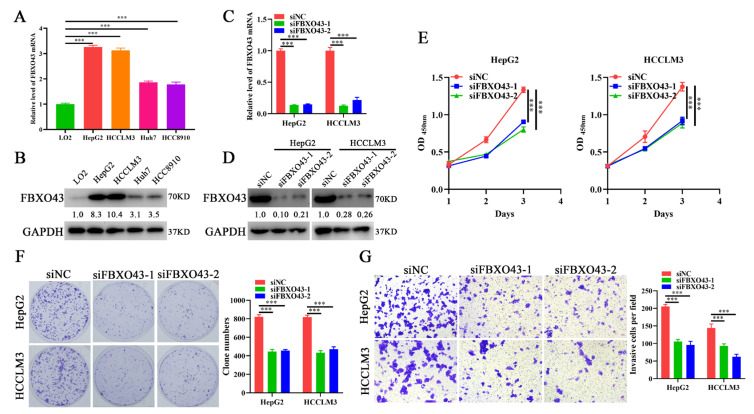
FBXO43 knockdown inhibits cell growth, proliferation and invasion in HCC. qRT-PCR (**A**) and WB (**B**) results indicating the mRNA and protein level of FBXO43 in normal liver cell and HCC cells. qRT-PCR (**C**) and WB (**D**) results indicating the mRNA and protein level of FBXO43 in HepG2 and HCCLM3 cells transfected with siFBXO43. CCK-8 (**E**), plate clone formation (**F**) and Transwell (**G**) assay showing the effects of FBXO43 depletion on the growth, proliferation and invasion of HepG2 and HCCLM3 cells. Magnification 100×, Scale bar 50 μm. ***, *p* < 0.001.

Data from TCGA, GTEx, and GENT2 databases revealed that FBXO43 expression was upregulated in both non-paired and paired HCC tissues (Figure 1A–D). Furthermore, the FBXO43 expression was significantly higher in HCC tissues with advanced T stage, late tumor grade, and higher serum alpha fetoprotein (AFP) level (Supplementary Figure S1A–C). Moreover, HCC patients with high FBXO43 expression had a poor prognosis in terms of overall survival (OS), disease-specific survival (DSS), progress-free interval survival (PFI), and relapse-free survival (RFS) (Figure 1E,F and Supplementary Figure S1D–E). The qRT-PCR, WB, and IHC assays confirmed FBXO43 upregulation in local HCC tissues and cells (Figure 1F,G and Figure 2A,B). The FBXO43 level was positively associated with high AFP level, advanced T stage, and WHO stage, as well as poor OS in HCC (Table 1 and Figure 1H). Thus, these findings reveal that FBXO43 is upregulated in HCC and has a positive correlation with advanced progression and poor prognosis.

### 3.2. FBXO43 Knockdown Inhibits the Proliferation and Invasion of HCC Cells

To investigate the functions of FBXO43 in HCC, siFBXO43s were used to knock out its expression in HepG2 and HCCLM3 cells. The results of qRT-PCR and WB (Figure 2C,D) confirmed the efficacy of FBXO43 knockdown in HCC cells. The effects of FBXO43 silencing on growth and invasion were determined by CCK-8, plate clone formation, and transwell invasion assays. The findings revealed that FBXO43 depletion significantly suppressed cell growth, proliferation, and invasion, evident from lower OD_450nm_ value, fewer clones, and invasive cells (Figure 2E–G). Therefore, these findings suggested that FBXO43 could promote HCC proliferation and invasion.

### 3.3. METTL3/IGF2BP2 Respectively Writes and Recognizes the m6A of FBXO43 mRNA and Enhances Its Stability in HCC

**Figure 3:**
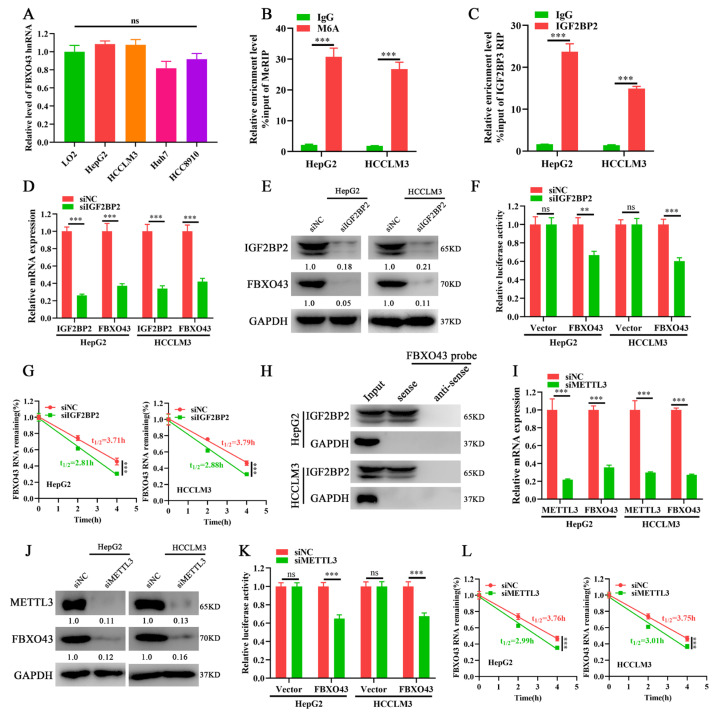
IGF2BP2 and METTL3 mediate FBXO43 M6A modification and maintain its stability in HCC. (**A**) qRT-PCR indicating the level of FBXO43 hnRNA between normal liver cell and HCC cells. (**B**) MeRIP-qPCR showing the m6A enrichment of FBXO43 transcripts in HepG2 and HCCLM3 cells. (**C**) RIP-qPCR demonstrating the enrichment of FBXO43 transcripts by IGF2BP2 immunoprecipitation in HepG2 and HCCLM3 cells. (**D**) qRT-PCR and (**E**) WB results indicating the effects of IGF2BP2 depletion on the level of FBXO43 transcript and protein in HepG2 and HCCLM3 cells. (**F**) Relative luciferase activity of HepG2 and HCCLM3 cells transfected with siNC and siIGF2BP2. (**G**) qRT-PCR indicating the stability of FBXO43 mRNA in HepG2 and HCCLM3 cells with IGF2BP2 depletion upon actinomycin D (10 μg/mL) treatment. (**H**) RNA pulldown assay showing the direct interaction of FBXO43 transcripts with the IGF2BP2 protein. (**I**) qRT-PCR and (**J**) WB results indicating the effects of METTL3 depletion on the level of FBXO43 transcript and protein in HepG2 and HCCLM3 cells. (**K**) Relative luciferase activity of HepG2 and HCCLM3 cells transfected with siNC and siMETTL3. (**L**) qRT-PCR indicating the stability of FBXO43 mRNA in HepG2 and HCCLM3 cells with METTL3 depletion upon actinomycin D (10 μg/mL) treatment. ns, no significant difference. **, *p* < 0.01; ***, *p* < 0.001.

The levels of FBXO43 hnRNAs, the primary transcripts of FBXO43 that indirectly indicates its transcription activity, were measured to investigate the mechanism of FBXO43 upregulation in HCC [18]. The qRT-PCR results (Figure 3A) demonstrated that the levels of FBXO43 hnRNAs were equivalent, implying that post-transcriptional mechanisms were responsible for FBXO43 upregulation in HCC. Considering the importance of m6A modification in the regulation of genes expression and the ENCORI data that IGF2BP2 knockdown, a critical m6A reader, significantly decreased FBXO43 expression in HepG2 cells (Supplementary Figure S2A). We further investigated whether the m6A-related mechanism was involved in the regulation of FBXO43 expression in HCC. Consequently, the SRAMP database predicted several very high-confidence m6A sites of FBXO43 mRNA [19] (Supplementary Table S2). Importantly, MeRIP-qPCR and RIP-qPCR results revealed that m6A and IGF2BP2 antibodies significantly enriched FBXO43 mRNA in HCC cells, respectively (Figure 3B,C), indicating that m6A modification of FBXO43, recognized by IGF2BP2, could regulate its expression in HCC. Indeed, IGF2BP2 expression was positively correlated with FBXO43 (Supplementary Figure S2B), and IGF2BP2 depletion significantly inhibited FBXO43 expression in HCC cells (Figure 3D,E). Furthermore, IGF2BP2 knockdown significantly reduced the stability of FBXO43 mRNA, as evidenced by dual-luciferase reporter and actinomycin D chase assay results (Figure 3F,G). Moreover, RNA pulldown assay results (Figure 3H) revealed that biotin-labeled FBXO43 mRNA probes specifically precipitated IGF2BP2 in HCC cells but did not control anti-sense probes. 

The writers mediated m6A of FBXO43 were then investigated. The TCGA data demonstrated that, among four common m6A writers (METTL3, METTL14, METTL16, and WTAP), METTL3 was upregulated, highly associated with FBXO43, and predicted poor prognosis in HCC (Supplementary Figure S2C–E). Therefore, METTL3 was selected for validation. In line with IGF2BP2 depletion, METTL3 silencing significantly reduced FBXO43 expression in HCC cells (Figure 3I,J). Moreover, METTL3 knockdown also significantly increased the turnover of FBXO43 mRNA in HCC, as evident by dual-luciferase reporter and actinomycin D chase assays (Figure 3K,L). Therefore, our findings revealed that the m6A reader IGF2BP2 interpreted METTL3-mediated FBXO43 m6A modification and maintained its stability in HCC.

### 3.4. FBXO43 Is an Effector of the Oncogenic Role of METTL3 and IGF2BP2 in HCC

**Figure 4:**
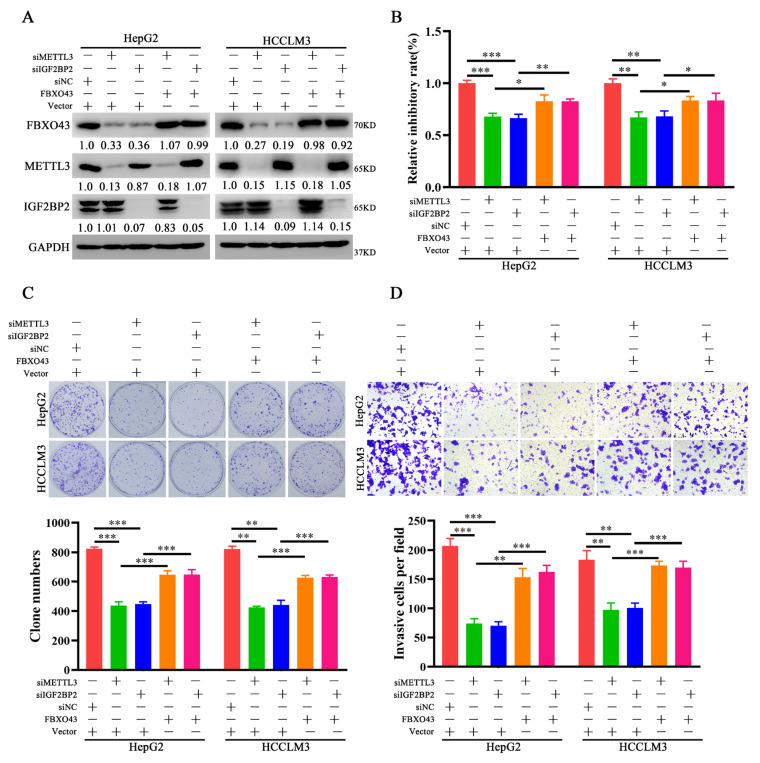
Ectopic expression of FBXO43 restores the effects of METTL3 and IGF2BP2 depletion in HCC. (**A**) WB results indicating the level of FBXO43, METTL3 and IGF2BP2 protein in HepG2 and HCCLM3 cells co-transfected with siMETTL3/siIGF2BP2 and FBXO43 plasmids. CCK-8 (**B**), plate clone formation (**C**,**D**) Transwell assay indicating the growth inhibitory, proliferation and invasion of HepG2 and HCCLM3 cells co-transfected with siMETTL3/siIGF2BP2 and FBXO43 plasmids. Scale bar 50 μm, * *p* < 0.05, **, *p* < 0.01; ***, *p* < 0.001.

Further, the role of FBXO43 in the oncogenic roles of METTL3 and IGF2BP2 in HCC was investigated. FBXO43 levels were restored in HCC cells after IGF2BP2 and METTL3 depletion, respectively, by ectopic expression of FBXO43 (Figure 4A). The proliferation and invasion of HCC cells were then investigated. The CCK-8, plate clone formation, and transwell invasion assay results (Figure 4B–D) revealed that ectopic FBXO43 expression significantly restored the growth and invasion abilities of HCC cells with METTL3 and IGF2BP2 knockdown, respectively. Therefore, our findings present that FBXO43 partially mediates the oncogenic roles of METTL3 and IGF2BP2 in HCC.

### 3.5. FBXO43 Promotes p53 Degradation by Maintaining UBE2C Expression in HCC

**Figure 5:**
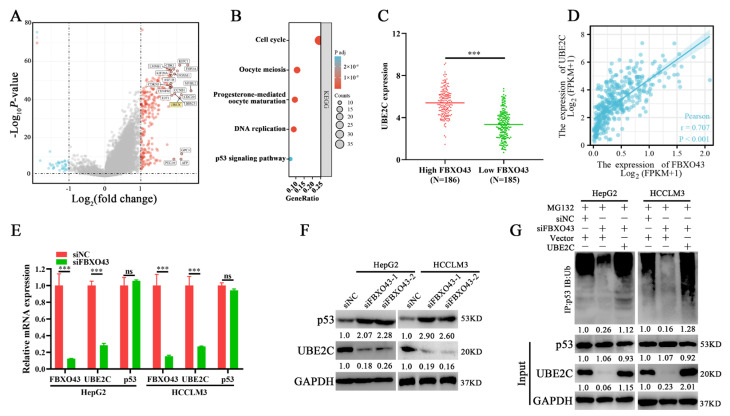
FBXO43 promotes proteasomal degradation of p53 via upregulating UBE2C. (**A**) Volcano plot indicating differentiated gene expression profiles between HCC tissues with low and high FBXO43 expression in TCGA. (**B**). The KEGG enrichment of differentiated genes between HCC tissues with low and high FBXO43 expression. (**C**) The expression of UBE2C in HCC tissues with low and high FBXO43. (**D**) The pearson correlation between FBXO43 and UBE2C expression in HCC tissues. (**E**) RT-qPCR indicating the expression of FBXO43, UBE2C, and p53 in HepG2 and HCCLM3 cells with FBXO43 depletion. (**F**) WB results indicating the level of UBE2C and p53 in HepG2 and HCCLM3 cells with FBXO43 depletion. (**G**) The IP and WB results showing the level of total ubiquitinated p53, p53, and UBE2C in HepG2 and HCCLM3 cells co-transfected with siFBXO43 and UBE2D expression plasmids. ns, no significant difference; ***, *p* < 0.001.

The differential expression profiles of HCC tissues with high and low FBXO43 expression were examined. The volcano plot (Figure 5A) and heat map (Supplementary Figure S3A) revealed 273 upregulated and 32 downregulated genes. The KEGG pathway enrichment analysis demonstrated that the differential genes were significantly enriched in cell cycle-related processes such as cell cycle, oocyte meiosis, and p53 signaling (Figure 5B). Particularly, several ubiquitin-conjugating enzymes, including UBE2C, UBE2T, and UBE2S that targeted p53 for degradation in cancers [20,21,22], were significantly upregulated in HCC tissues with high FBXO43 and positively correlated with FBXO43 expression in HCC tissues (Figure 5C,D and Supplementary Figure S3B,C). Subsequently, qRT-PCR results confirmed that FBXO43 depletion significantly downregulated UBE2C expression but not p53, UBE2T, and UBE2S (Figure 5E and Supplementary Figure S3D). Then, WB results revealed that FBXO43 knockdown downregulated UBE2C while upregulated p53 levels in HCC cells (Figure 5F), implying that FBXO43 promoted p53 degradation via UBE2C. Indeed, FBXO43 depletion significantly reduced the p53 total ubiquitination level, which could be reversed by ectopic expression of UBE2C in HCC cells with FBXO43 knockdown (Figure 5G). Besides, the expression of p21, a known target of p53 [22], and cleaved-Parp were detecteted in HCC cells with FBXO43 knockdown. The results (Supplementary Figure S4A,B) indicated that FBXO43 depletion significantly upregulated p21 expression and cleaved-Parp level, suggesting FBXO43 suppressed p53 activity via promoting its degradation. Therefore, these findings revealed that FBXO43 promoted p53 degradation by upregulating UBE2C expression in HCC.

### 3.6. FBXO43 Exerts Oncogenic Roles by Regulating the UBE2C/p53 Axis in HCC

**Figure 6:**
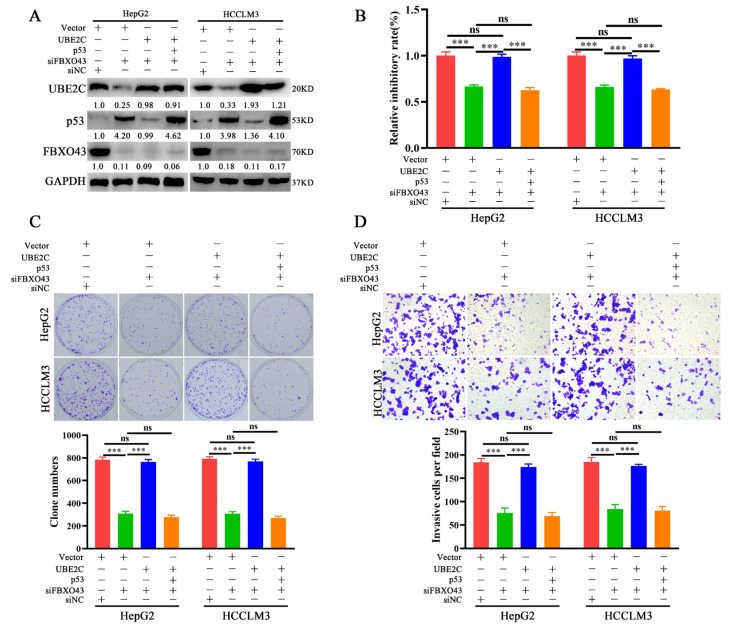
FBXO43 promotes cell proliferation and invasion via UBE2C/p53 axis in HCC. (**A**) WB results indicating the level of UBE2C, p53 and FBXO43 in HepG2 and HCCLM3 cells co-transfected with siFBXO43, UBE2C and p53 expression plasmids. CCK-8 (**B**), plate clone formation (**C**,**D**) Transwell assay indicating the growth inhibitory, proliferation and invasion of HepG2 and HCCLM3 cells co-transfected with siFBXO43, UBE2C and p53 expression plasmids. Scale bar 50 μm; ns stands for no significant difference; ***, *p* < 0.001.

The role of the UBE2C/p53 axis in oncogenic functions was investigated further in HCC. In HCC cells with FBXO43 knockdown, ectopic expression of UBE2C re-decreased the level of p53, which could be recovered by ectopic expression of p53 again, as represented in Figure 6A. Therefore, the findings of functional experiments such as CCK-8, plate clone formation, and transwell invasion assays revealed that UBE2C overexpression could significantly remove the inhibitory effects of FBXO43 depletion on cell proliferation and invasion, which were re-suppressed by co-overexpression of p53 and UBE2C in HCC cells with FBXO43 knockdown (Figure 6B–D). 

Considering the relative high mutant frequency of p53 in HCC [23], the expression of FBXO43 between p53-wild and -mutant HCC tissues was also analyzed. As the Supplementary Figure S4C shown, compared with normal liver tissues, FBXO43 was upregulated in HCC tissues bearing both wild and mutant p53. Notably, FBXO43 was highly expressed in p53-mutant HCC tissues than that in p53-wild HCC tissues (Supplementary Figure S4C), suggesting that p53 may involve in regulation of FBXO43 in HCC. Thus, we detected the effect of p53 overexpression on FBXO43 in HCC cells. The results (Supplementary Figure S4D) indicated that p53 overexpression did not alter the expression of FBXO43 in HCC cells, suggesting the absence of feedback between p53 and FBXO43 in HCC. Moreover, FBXO43 knockdown also significantly suppressed cell growth and invasion of Huh7 cells (Supplementary Figure S5A–C), which expressed Y220C-mutated p53 without transcription activity [24], suggesting the FBXO43 exerted consistently oncogenic roles in both p53-wild and -mutant HCC cells. Of note, the detail oncogenic mechanism of FBXO43 in p53-mutant HCC cells remains to be further investigated.

**Figure 7:**
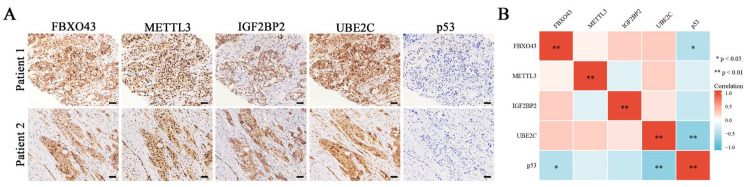
The co-expression of M6A regulators/FBXO43/UBE2C/p53 in HCC tissues. (**A**) The representative pictures of co-expression of FBXO43, METTL3, IGF2BP2, UBE2C, and p53 staining in the same HCC tissues. (**B**) The heatmap indicating the correlation and statistical significance of M6A regulators/FBXO43/UBE2C/p53 in HCC tissues (*n* = 20). Scale bar 100 μm; *, *p* < 0.05, **, *p* < 0.01.

At last, the IHC results confirmed the negative correlations of p53 between FBXO43 and UBE2C in HCC tissues (Figure 7A,B). Thus, these findings demonstrated that the UBE2C/p53 axis at least partially mediated the oncogenic functions of FBXO43 in HCC.

## 4. Discussion

Although the role of FBXO43 in biological processes has been primarily revealed in the negative regulation of meiosis II metaphase [2,3], the FBXO43 upregulation and oncogenic functions in carcinogenesis have recently been reported in several cancers. For example, FBXO43 upregulation is associated with malignant clinical features such as tumor grade, tumor size, and lymph node metastasis and may predict poor prognosis in breast cancer patients [6]. Downregulation of FBXO43 has been shown to significantly suppress the malignant phenotypes of breast cancer cells, which may be dependent on proliferating cell nuclear antigen (PCNA) degradation and inactivation [5]. Bioinformatics analysis revealed that FBXO43 was upregulated in gastric cancer and HCC [7,14]. In line with these studies, our findings experimentally confirmed the upregulation and oncogenic functions of FBXO43 in HCC, which was also associated with advanced clinical progression and predicted poor prognosis in HCC patients.

Neither the transcriptional nor post-transcriptional mechanisms that regulate FBXO43 expression have been identified. Because hnRNAs are the primary products of gene transcription, their levels indicate the transcriptional activity of the coded genes [18,25]. Therefore, detecting the hnRNAs level is an effective way to distinguish between transcriptional and post-transcriptional mechanisms. In the present study, the levels of FBXO43 hnRNA in normal liver cells and HCC cells are comparable, implying that post-transcriptional mechanisms are responsible for FBXO43 upregulation In HCC. 

m6A is a common post-transcriptional mechanism that regulates the stability, translation, alternative splicing, and subcellular localization of tumor-related substrate RNAs [9,10]. The critical roles of m6A regulators in multiple cancers, especially in HCC, have been well summarized in recent reviews [26,27]. For example, METTL3 is upregulated and predicts poor prognosis in HCC, while METTL3 depletion significantly suppresses HCC growth both in vitro and in vivo by eliminating SOCS2 m6A modification and subsequently upregulating SOCS2 expression [28]. METTL16 upregulation is associated with a poorer outcome in HCC patients and promotes malignant progression by promoting lncRNA RAB11B-AS1 degradation in an m6A-dependent manner [29]. Therefore, the oncogenic roles of IGF2BP2 in HCC largely depend on the interpretation of m6A of oncogenes like c-Myc, FEN1, and CDC27 [30,31,32]. The present study validated the oncogenic roles of METTL3 and IGF2BP2 in HCC. We also identified that METTL3 and IGF2BP2 mediated the m6A of FBXO43 and stabilizeD FBXO43 mRNA, which accounted for its upregulation and subsequently facilitated the METTL3 and IGF2BP2 oncogenic functions in HCC. 

A well-known tumor suppressor, p53, is regulated by multiple mechanisms in cancers, including both transcriptional and post-transcriptional mechanisms [33,34,35]. Notably, ubiquitin-dependent proteasomal degradation is a critical pathway in p53 regulation [34,35]. Ubiquitin E3 ligases, such as MDM2 [36], TRIM32 [37], and RNF187 [38], exert their oncogenic roles by targeting p53 for proteasomal degradation. Apart from oncogenic ubiquitin E3 ligases, the tumorigenic capacities of several ubiquitin-conjugating enzymes (E2) also promote proteasomal degradation of p53, particularly in HCC. For example, overexpressed ubiquitin-conjugating enzymes, such as UBE2C [20], UBE2T [21], and UBE2S [22], directly bind and trigger proteasomal degradation of p53, promoting malignant progression in HCC. Moreover, we also identified that FBXO43 could indirectly promote p53 proteasomal degradation in HCC by upregulating UBE2C expression, which largely accounts for the oncogenic function of FBXO43 in HCC.

Unfortunately, the mechanism by which FBXO43 regulates UBE2C expression in HCC remained unidentified in the present study. Transcriptional and epigenetic regulators, such as transcription factors and micro RNAs, have been identified to regulate UBE2C in cancers [39,40,41]. For example, FOXM1 is a validated transcription factor of UBE2C in esophageal squamous cell carcinoma [39]. Although FOXM1 is upregulated in HCC tissues with high FBXO43, FBXO43 depletion did not change FOXM1 expression in HCC cells, implying that FOXM1 plays little role in UBE2C regulation by FBXO43. Moreover, although FBXO43 was differentially expressed in p53-wild and mutant HCC tissues, FBXO43 was not regulated by p53 and consitently exert oncogenic roles in both p53-wild and mutant HCC cells, which demanded more efforts to reveal the underlying mechanisms in the following work.

## 5. Conclusions

The present study confirmed the upregulation, prognostic value, and oncogenic roles of FBXO43 in HCC. The METTL3 and IGF2BP2-mediated m6A modification stabilized and upregulated FBXO43, facilitating the malignant progression of HCC. FBXO43 upregulated UBE2C expression, which then triggered ubiquitin-dependent proteasomal degradation of p53 to promote tumorigenesis in HCC. Therefore, the present study revealed the upregulation and oncogenic roles of FBXO43, as well as the mechanism underlying the upregulation and oncogenic role of FBXO43, emphasizing the potential application of FBXO43/UBE2C/p53 axis in HCC targeted therapy.

## Figures and Tables

**Table 1:** Correlation of FBOX43 expression with clinicopathologic features in HCC tissues (Fisher’s exact test, *n* = 80).

Variables	N	FBOX43 Expression		*p* Value
		High	Low	
Age				
≤45	38	28	10	>0.9999
>45	42	31	11	
Sex				
Male	75	54	21	0.3184
Female	5	5	0	
AFP (ng/mL)				
≤400	38	20	18	<0.0001
>400	42	39	3	
T stage				
1	18	9	9	0.0148
2 + 3	62	50	12	
Hep-1 staining				
Positive	61	44	17	0.7665
Negative	19	15	4	
Tumor diameter				
≤6cm	37	26	11	0.6127
>6cm	43	33	10	
WHO stage				
II	11	5	6	0.0317
III-IV	69	54	15	

Variables

N

FBOX43 Expression

*p* Value

High

Low

## Data Availability

Publicly available data were used in this study. The raw data used are available from TCGA (https://portal.gdc.cancer.gov/, accessed on 10 October 2022). The rest data needed to evaluate the conclusions of this paper are present in the paper and/or Supplementary Materials. Additional data related to this paper may be requested from the correspondence author.

## Supplementary Materials

The following supporting information can be downloaded at: https://www.mdpi.com/article/10.3390/cancers15030957/s1, Figure S1: The expression of FBXO43 in subtype of HCC and its prognostic value based on Kaplan-Meier Plotter data. Figure S2: The online analyses of correlation with FBXO43 and prognostic value of M6A writers. Figure S3: FBXO43 positively correlates with UBE2T and UBE2S, but does not regulate their expression. Figure S4: Analyzing the relaiton between FBXO43 and p53 status in HCC. Figure S5: FBXO43 knockdown inhibits cell growth and invasion in Huh7 cells. Table S1: The sequences of primers and siRNAs; Table S2: The M6A sites of FBXO43 predicted by SRAMP database.
