# Supplementary material for: F-Box Protein 43, Stabilized by N6-Methyladenosine Methylation, Enhances Hepatocellular Carcinoma Cell Growth and Invasion via Promoting p53 Degradation in a Ubiquitin Conjugating Enzyme E2 C-Dependent Manner
Source: Cancers (Basel). 2023 Feb 2;15(3):957. doi: 10.3390/cancers15030957 (PMC9913344; doi:10.3390/cancers15030957)

Supplementary Figure legends

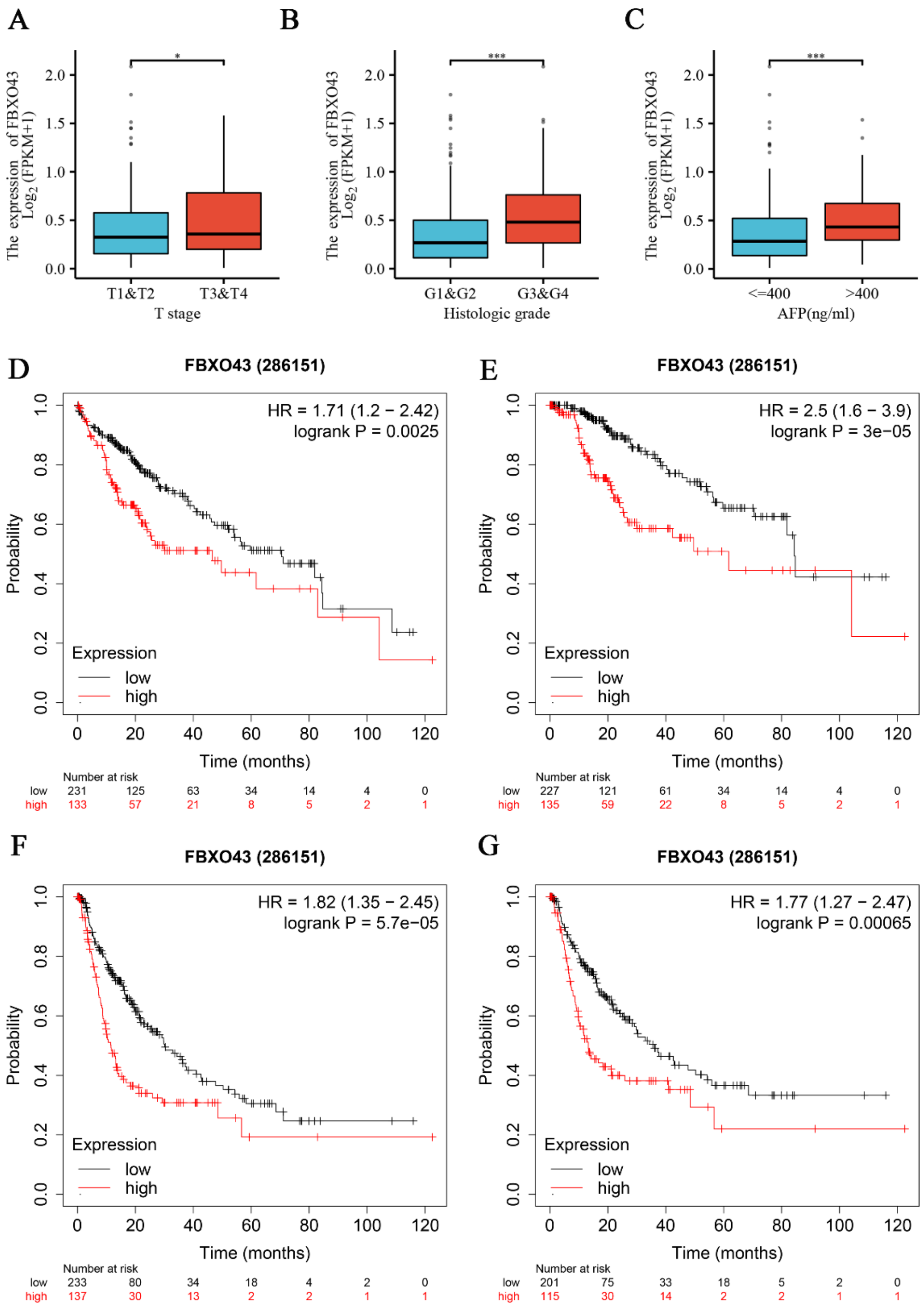

Supplementary Figure S1. The expression of FBXO43 in subtype of HCC and its prognostic value based on Kaplan-Meier Plotter data. The expression of FBXO43 in subtype of HCC patients: (A) stage T1 plus T2 vs T3 plus T4; (B) grade G1 plus G2 vs G3 plus G4; (C) AFP (≤400 ng/ml) vs AFP (>400 ng/ml). The survival curves from Kaplan-Meier Plotter indicating the prognostic value of FBXO43 including OS (**D**),

relapsing free survival (**E**), progression free survival (**F**), and DSS (**G**), * p < 0.05, ***, p < 0.001.

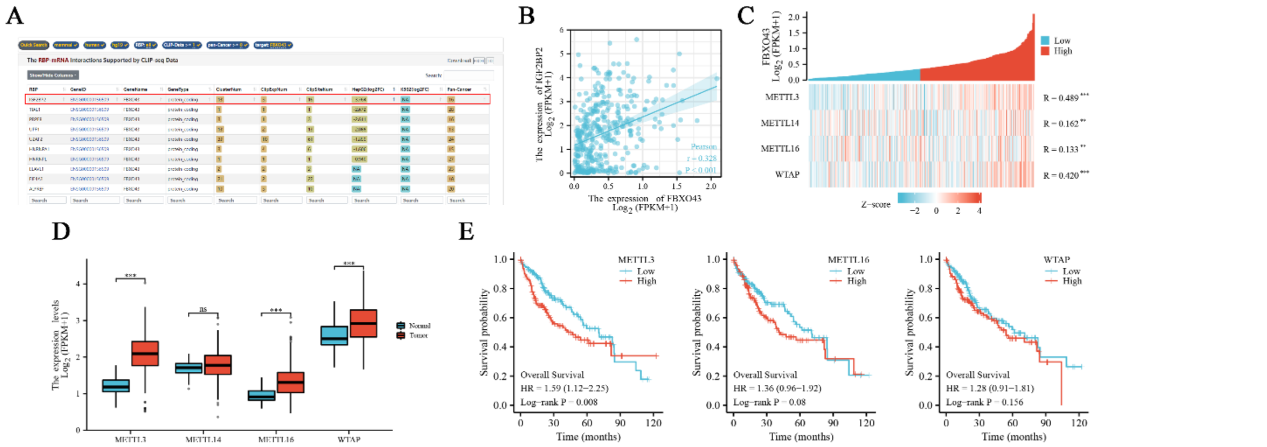

Supplementary Figure S2. The online analyses of correlation with FBXO43 and prognostic value of M6A writers. (**A**) ENCORI data showing IGF2BP2 could bind FBXO43 and negatively regulate its expression in HepG2 cell. (**B**) and (**C**)The TCGA data indicating the correlation between IGF2BP2 and M6A writers, including METTL3, METTL14, METTL16, and WTAP, HCC. (**D**) and (**E**) The TCGA data indicating the expression and prognostic value of M6A writers in HCC. **, p < 0.01,***, $P < 0.001$; **ns**, no significant difference.

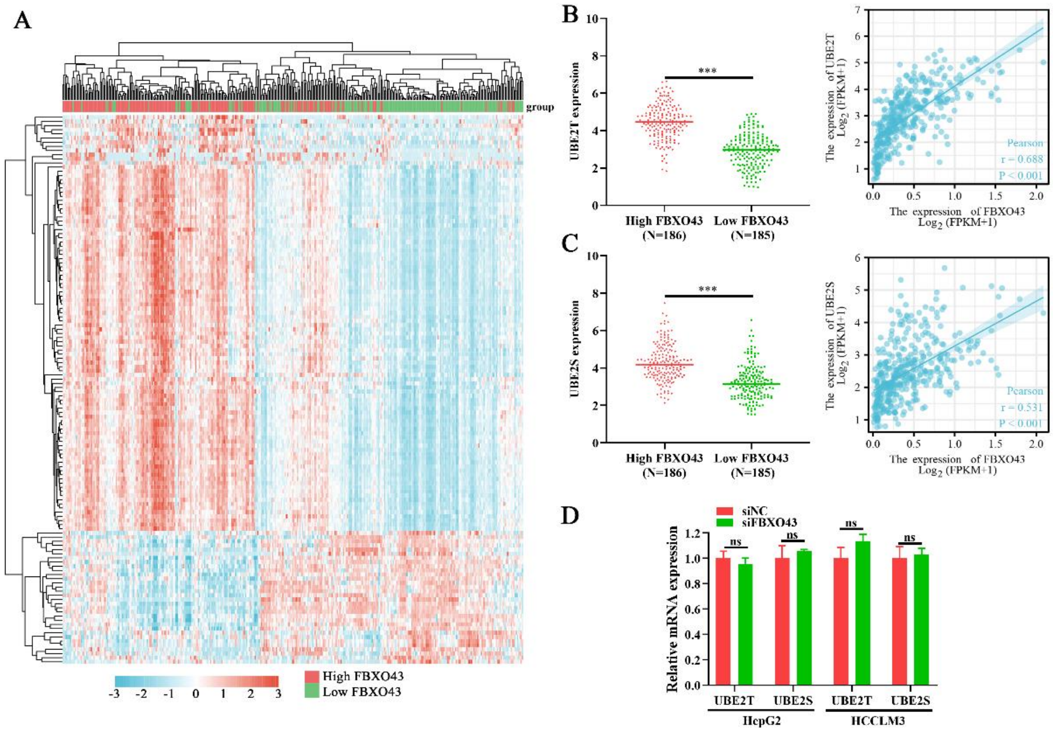

Supplementary Figure S3. FBXO43 positively correlates with UBE2T and UBE2S, but does not regulate their expression. (**A**) Heap-map of differentiated genes between

HCC tissues with low and high FBXO43 expression in TCGA. (**B**) The expression of UBE2T in HCC tissues with low and high FBXO43 and its correlation with FBXO43 in HCC tissues. (**C**) The expression of UBE2S in HCC tissues with low and high FBXO43 and its correlation with FBXO43 in HCC tissues. (**D**) RT-qPCR indicating the expression of UBE2T and UBE2S in HepG2 and HCCLM3 cells with FBXO43 depletion. ***, $P < 0.001$; **ns**, no significant difference.

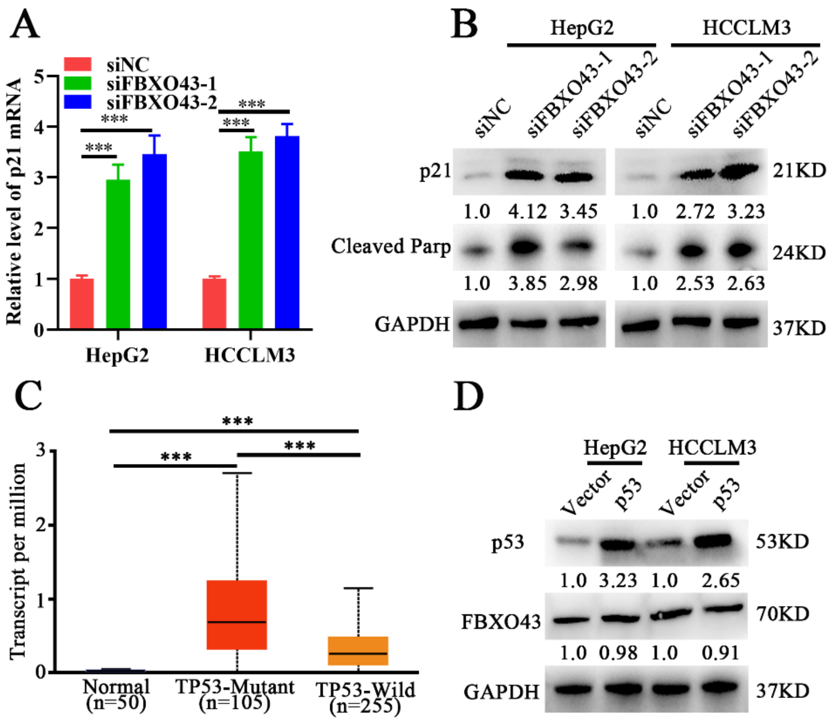

Supplementary Figure S4. Analyzing the relaiton between FBXO43 and p53 status in HCC. (**A**) RT-qPCR indicating the expression of p21 in HepG2 and HCCLM3 cells with FBXO43 depletion. (**B**) WB results indicating the level of p21 and cleaved-Parp in HepG2 and HCCLM3 cells with FBXO43 depletion (**C**) The UALCAN data showing the expression of TP53 among normal, TP53-mutant, and TP52- wild HCC tissues. (**D**) WB results indicating the level of p53 and FBXO43 in HepG2 and HCCLM3 cells with p53 overexpression. ***, $P< 0.001$.

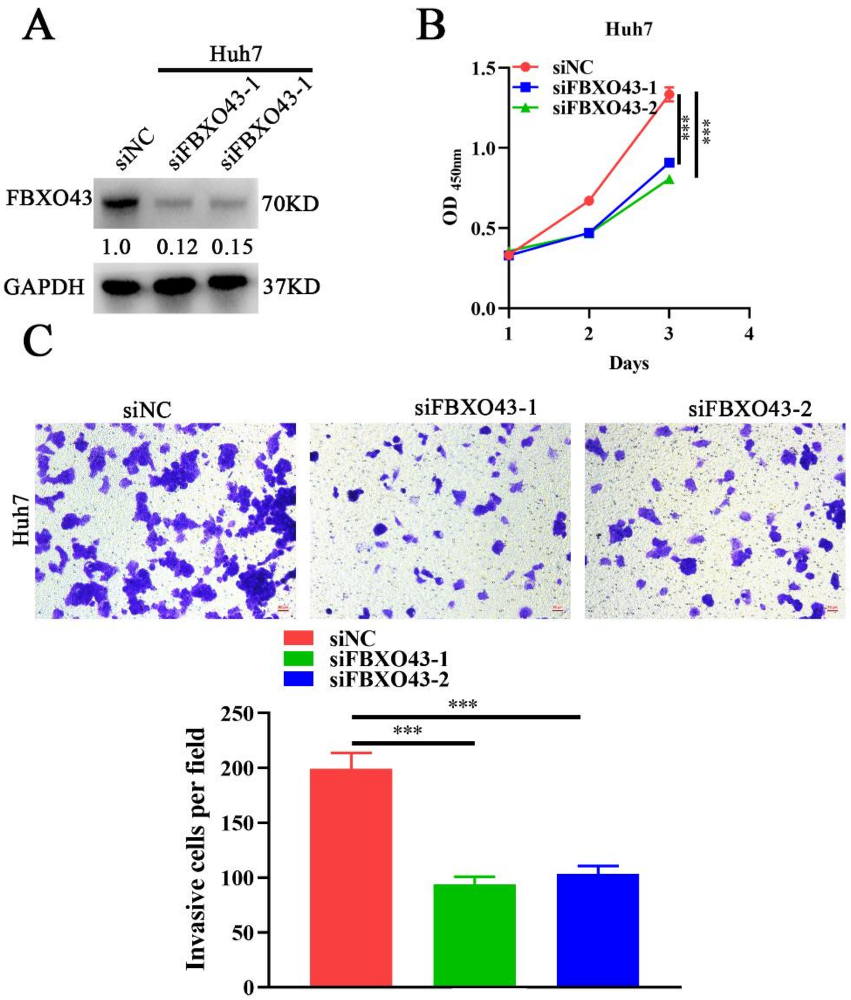

Supplementary Figure S5. FBXO43 knockdown inhibits cell growth and invasion in Huh7. (**A**)WB results indicating the protein level of FBXO43 in Huh7 cells transfected with siFBXO43s. qRT-PCR. CCK-8 (**B**) and Transwell (**C**) assay showing the effects of FBXO43 depletion on the growth and invasion of Huh7 cells. Magnification 100×, Scale bar 50μm. ***, $P < 0.001$.

Supplementary Table S1. The sequences of primers and siRNAs

Names	Sequences(5'-3')
**FBXO43**	
Forward	GGAAAGTAAGCAGAAATTGGCGTG
Reverse	GAGTGGCAGCATCCTCGACATT
**FBXO43 hnRNA**	
Forward	CAGTTGCGAAGAACCACGTTT
Reverse	AGTTGCCTCCAACGTCCTTT
**IGF2BP2**	
Forward	AGTGGAATTGCATGGGAAAATCA
Reverse	CAACGGCGGTTTCTGTGTC
**METTL3**	
Forward	TTGTCTCCAACCTTCCGTAGT
Reverse	CCAGATCAGAGAGGTGGTGTAG
**UBE2C**	
Forward	CTGGCGATAAAGGGATTTCTGCC
Reverse	GCGAGAGCTTATACCTCAGGTC
**UBE2T**	
Forward	AGCTGCTCATGTCAGAACCCAAC
Reverse	GTCTGGCATTCTTGAGGAAGGC
**UBE2S**	
Forward	CCGACACGTACTGCTGACC
Reverse	GCCGCATACTCCTCGTAGTTC
**p53**	
Forward	CCTCAGCATCTTATCCGAGTGG
Reverse	TGGATGGTGGTACAGTCAGAGC
**p21**	
Forward	TGTCCGTCAGAACCCATGC
Reverse	AAAGTCGAAGTTCCATCGCTC
**GAPDH**	
Forward	CAGCAAGAGCACAAGAGGAA
Reverse	ATGGTACATGACAAGGTGCGG
**T7-FBOX43 sense**	
Forward	TAATACGACTCACTATAGGGATGAGTTTTAAAGACAAAGAT
Reverse	GAGGCGTTTTAAATTCCGCTT
**T7-FBOX43 anti-sense**	
Forward	TAATACGACTCACTATAGGGGAGGCGTTTTAAATTCCGCTT
Reverse	ATGAGTTTTAAAGACAAAGATG
**siFBOX43-1**	CGTGAAATTGTTGTTCAAGAT
**siFBOX43-2**	GCCAAGTTATCAACTTAGAAA
**siIGF2BP2**	AGTGAAGCTGGAAGCGCATAT
**siMETTL3**	GCCAAGGAACAATCCATTGTT

Supplementary Table S2. The M6A sites of FBXO43 predicted by SRAMP database

Position	Sequence context	Score (binary)	Score (knn)	Score (spectrum)	Score (combined)	Decision(site confidence)
837	ACTGAAGCAGGAAATGGGGCGGA<br>CTCTCCTCCAATTGTCAACTCC	0.647	0.731	0.517	0.599	High
1102	AGGAAAAGGATAAAACCCCAGAA<br>CTTTGTGAAACACCTAAAATCA	0.718	0.634	0.593	0.664	Very high
1185	TCTTTCGCTCTTCTAAAAGGGGAC<br>TTTGAATCACAAAATAGTTCT	0.763	0.879	0.549	0.683	Very high
1331	CACTTTAAAAACAGAAGAAGTGA<br>CTTCATGCAGTCAAAAATTGAG	0.566	0.699	0.582	0.579	Moderate
1376	GCTTAATTTTTCTCAGCAAAAGAC<br>TTCCACAATTGATGATTCCAA	0.619	0.758	0.496	0.577	Moderate
1461	ATTCAGGGCAATAATTTTAAAGACT<br>CTATCACACATGACTTTAGT	0.661	0.723	0.480	0.592	Moderate
1753	ACCAGGAGGGTTCTTTTCAAGAAC<br>TACTGCAGAAACATAAGGGGA	0.563	0.498	0.603	0.576	Moderate
1775	ACTACTGCAGAAACATAAGGGGAC<br>TCCCAAAGTTGGGGACACCAT	0.639	0.696	0.613	0.631	High
1791	AAGGGGACTCCCAAAGTTGGGGA<br>CACCATAAGAAAGACAAGACAT	0.581	0.418	0.611	0.585	Moderate
1831	GACATCTTGGAAGGTCGAGAAGA<br>CTGTCCACCCTTCGGGAACAAA	0.578	0.507	0.516	0.550	Low
2134	TACTGCAGTGTATACTTGCAGGAC<br>TGATCGGCAAGAAAATGGGTA	0.730	0.797	0.645	0.699	Very high

2164	GCAAGAAAATGGGTATAGAAAAAC TGGACATCTTAACAGAATTAA	0.578	0.644	0.674	0.620	High
2169	AAAATGGGTATAGAAAAACTGGAC ATCTTAACAGAATTAAAATAT	0.631	0.765	0.653	0.646	High
2650	ACCAGCCATATAAGAAAAGGGGAC TGTGTAGCCGAACAGCCTGTG	0.759	0.606	0.315	0.574	Moderate
2814	AATTTAAAACGCCTCTGAAGAGAC TAAATATAGAACTCCCCCATG	0.659	0.547	0.658	0.653	Very high
2826	CTCTGAAGAGACTAAATATAGAAC TCCCCCATGCAGTGTTCTATT	0.561	0.536	0.669	0.603	High
3173	CAGATAATATTAAAGATTTTGAACT CTAGACAGATGGCCTTATAG	0.691	0.685	0.567	0.641	High
3216	AGATTTGGAAAATGAGTACTTGAC TGAAATGAAATATAAATAAAA	0.624	0.689	0.570	0.606	High

Both uncropped exposure and white pictures were offered as follow:

Figure 2B

FBXO43

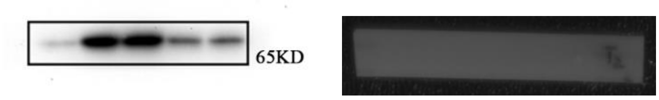

GAPDH

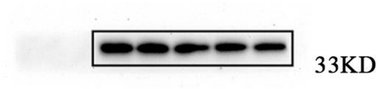

Figure 2D

FBXO43

Left panel:

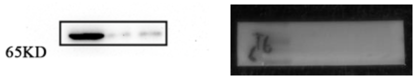

Right panel:

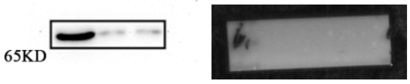

GAPDH

Left panel:

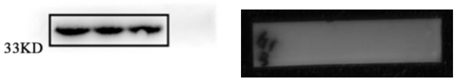

Right panel:

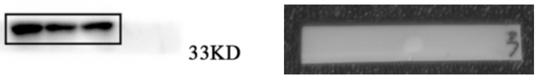

Figure 3E

IGF2BP2

Left panel:

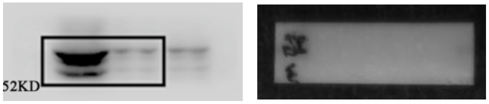

Right panel:

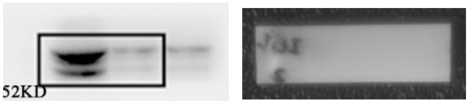

FBXO43

Left panel:

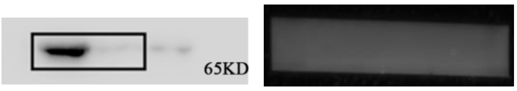

Right panel:

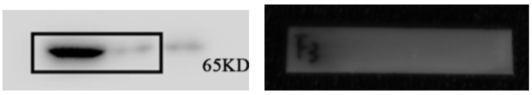

GAPDH

Left panel:

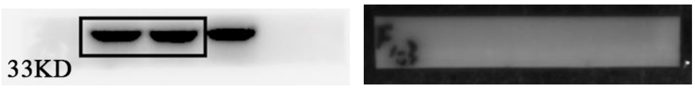

Right panel: 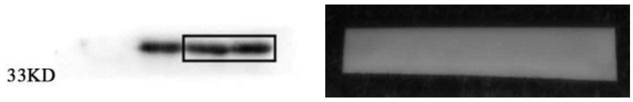

Figure 3H

IGF2BP2

Upper panel: 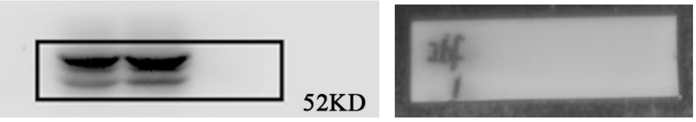

Lower panel: 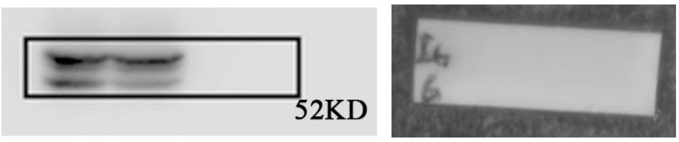

GAPDH

Upper panel: 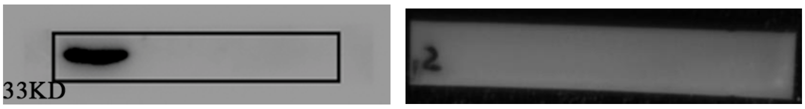

Lower panel: 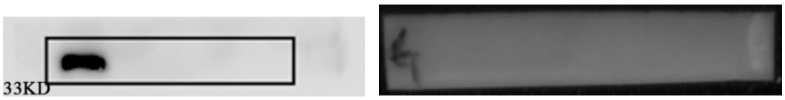

Figure 3J

METTL3

Left panel: 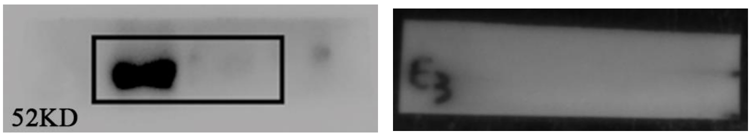

Right panel: 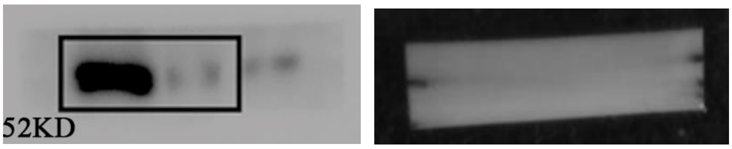

FBXO43

Left panel: 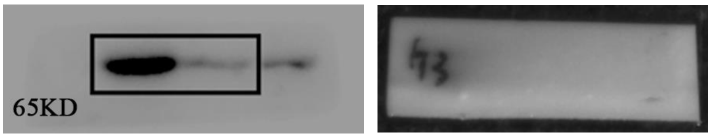

Right panel: 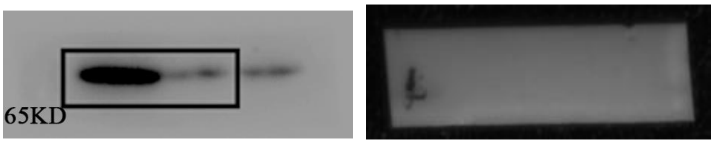

GAPDH

Left panel: 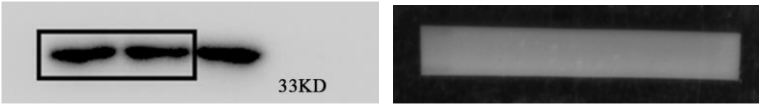

Right panel: 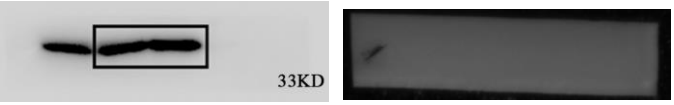

Figure 4A

FBXO43

Left panel: 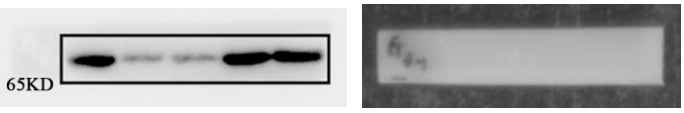

Right panel: 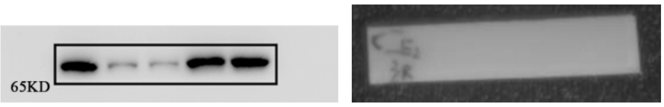

METTL3

Left panel: 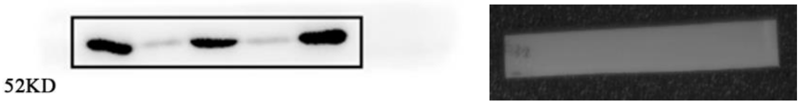

Right panel: 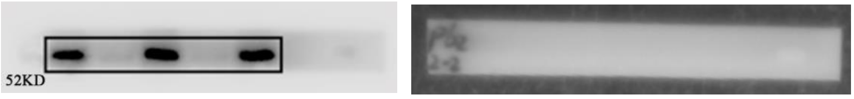

IGF2BP2

Left panel: 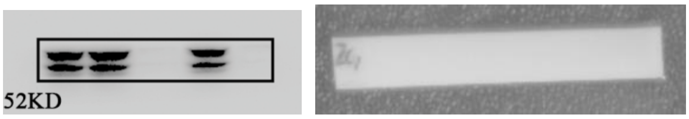

Right panel: 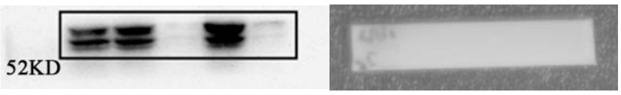

GAPDH

Left panel: 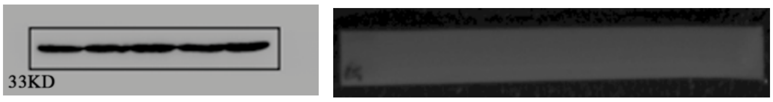

Right panel: 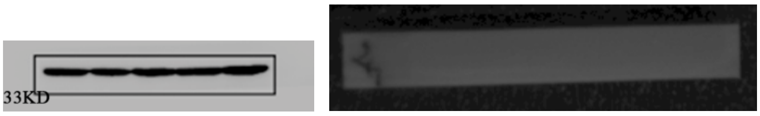

Figure 5F

p53

Left panel: 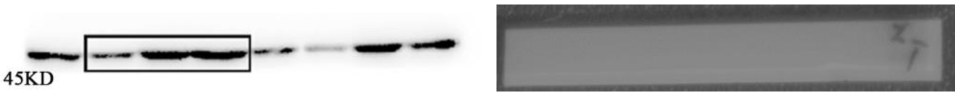

Right panel: 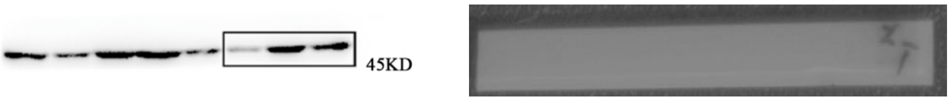

UBE2C

Left panel:

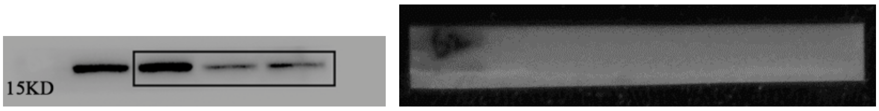

Right panel:

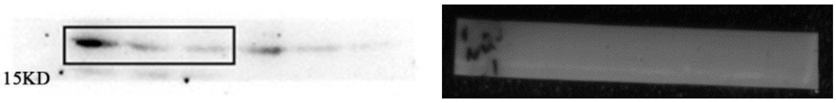

GAPDH

Left panel:

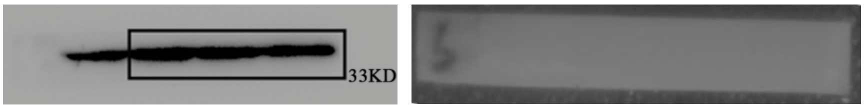

Right panel:

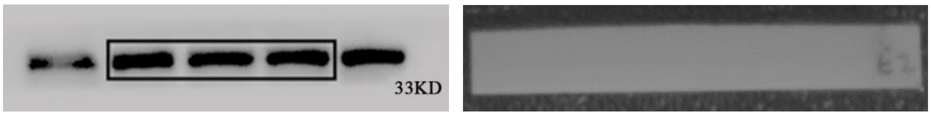

Figure 5G

IP

Left panel:

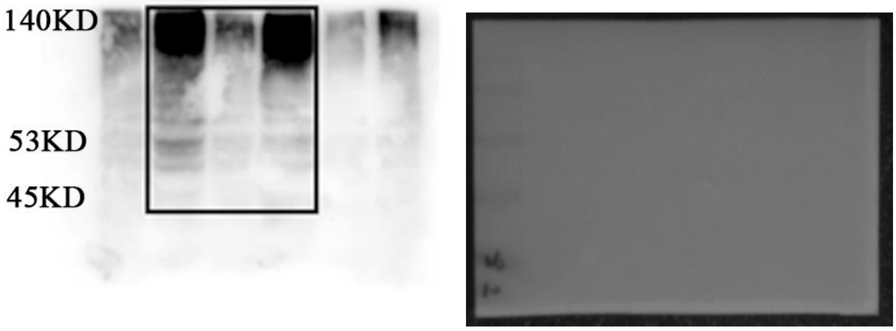

Right panel:

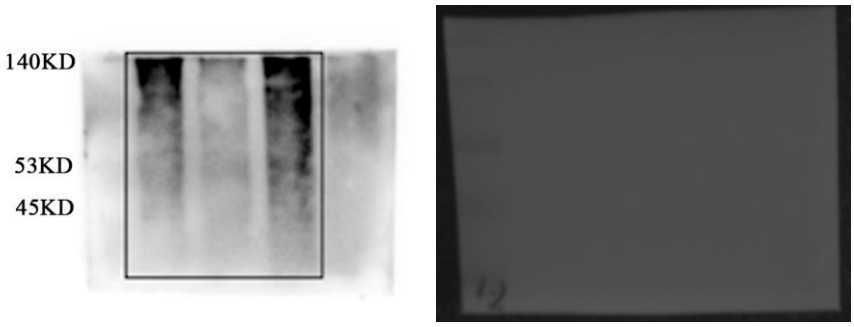

p53

Left panel:

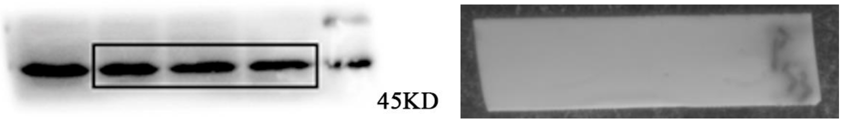

Right panel:

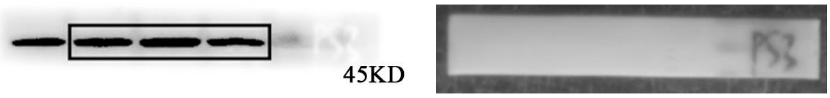

UBE2C

Left panel:

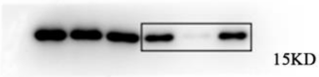

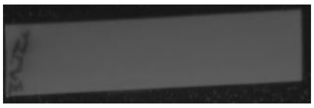

Right panel:

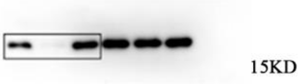

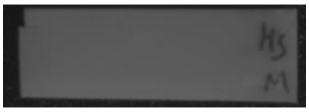

GAPDH

Left panel:

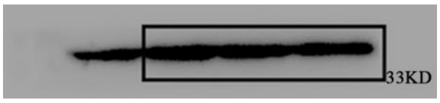

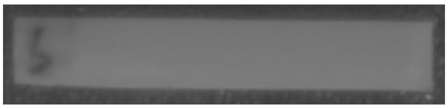

Right panel:

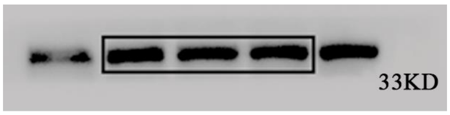

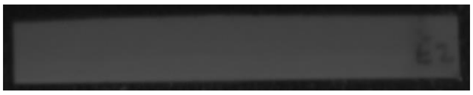

Figure 6A

UBE2C

Left panel:

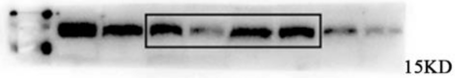

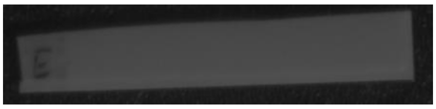

Right panel:

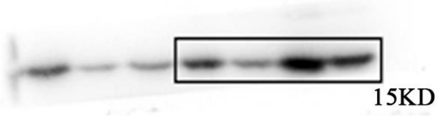

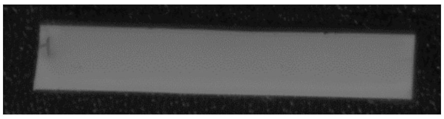

p53

Left panel:

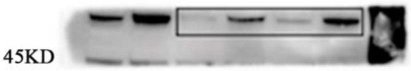

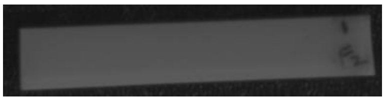

Right panel:

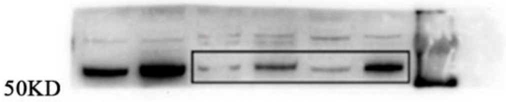

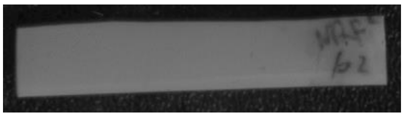

FBXO43

Left panel:

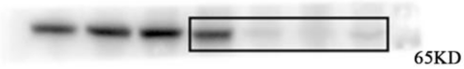

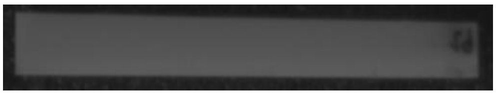

Right panel:

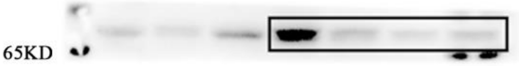

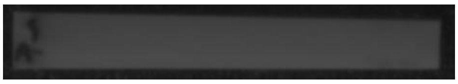

GAPDH

Left panel:

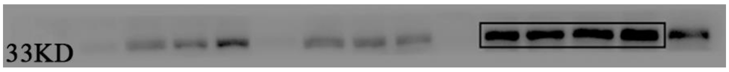

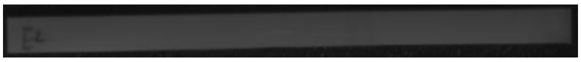

Right panel:

33KD

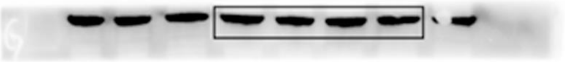

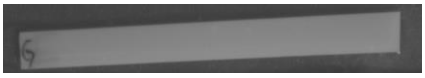

Supplement: Supplementary file 1 [file cancers-15-00957-s001.zip › cancers-2132595-supplementary.pdf]
